# Marginal Adaptation of Veneers: A Systematic Review

**DOI:** 10.7759/cureus.31885

**Published:** 2022-11-25

**Authors:** Vijetha Badami, Mittapalli Satya Priya, Linju Vijay, Hemabhanu Kethineni, Sneha Akarapu, Shruti Agarwal

**Affiliations:** 1 Department of Conservative Dentistry and Endodontics, MNR Dental College and Hospital, Hyderabad, IND

**Keywords:** cad-cam milling, systematic review, computer-aided design, dental veneers, dental marginal adaptation

## Abstract

This study aimed to systematically review the literature to evaluate the marginal adaptation of veneers using different fabrication methods, namely, conventional feldspathic porcelain laminate veneers (PLVs), computer-aided design-computer-aided machining (CAD-CAM) veneers, and pressed veneers. A comprehensive literature search was performed using electronic databases (PubMed and Google Scholar) as well as hand searches to identify all relevant studies related to veneers and marginal adaptation. The identified studies were screened for assessing the inclusion and exclusion criteria. The included articles were then subjected to data extraction and analysis. The search resulted in 130 articles, of which six were included in this systematic review. All included articles were assessed for adaptation of margins. Based on the findings of this systematic review, no significant differences were found in the marginal adaptation of CAD-CAM and conventional feldspathic PLVs. The marginal fidelity of ceramic veneers issuing from the various fabrication techniques was clinically acceptable.

## Introduction and background

Well-aligned teeth and color are the two most important aspects of an attractive smile. Patients’ interest in the treatment of their smile is steadily growing. Similarly, the treatment options to restore the esthetic appearance have also been increasing. For a long period in the past, the most durable and predictable treatment for esthetically compromised teeth was achieved by the preparation of a full crown. With the increase in the trend toward tooth conservation, bonding, and minimally invasive procedures, the interest in veneers has also been increasing.

John Calamia in the 1980s launched porcelain laminate veneers at New York University, United States [1]. Veneers are thin-bonded ceramic restorations that involve the labial surface and part of the proximal surfaces of anterior teeth that require esthetic corrections [2]. They offer several advantages such as excellent esthetics, superior biocompatibility, and durability.

Porcelain veneers provide a conservative treatment option for discolored and malformed vital anterior teeth. The indications include moderate discoloration caused by tetracycline stains, excessive fluoride intake, developmental malformations such as peg laterals, amylogenesis imperfecta, and diastema correction.

A critical factor for successful restoration is its marginal fit. The circumferential periphery of the prepared tooth is termed the finish line. Optimal preparation design and choice of restorative material enhance marginal adaptation and fracture resistance for long-term success [3]. The marginal gap is the perpendicular distance from the internal surface of the restoration to the finish line of the preparation [4]. Because veneers are bonded by resin cement, they become a consolidated portion of the tooth and bear the brunt of masticatory forces, temperature alterations, and hydrolytic disintegration by chemical and moisture contamination. Intimate proximity between the veneer tooth interface fortifies the resin cement from unrestrained exposure to oral conditions. A veneer with inferior marginal adaptation can mutilate the tooth, periodontal tissue leading to microleakage, and plaque accumulation, resulting in caries, pulpal lesions, gingival inflammation, and periodontal disease. A restoration with poor marginal fit can damage the tooth, periodontal tissue, and even restoration. These marginal discrepancies can lead to cement dissolution, microleakage, and plaque accumulation, which result in gingival inflammation, caries, and pulpal lesions.

Accurate marginal adaptation of an indirect restoration plays a significant role in periodontal health, with irregular or rough margins irritating the gingiva. The luting material is the weakest restorative link, and the dissolution of the cement can create a marginal gap and space for bacteria. Although a consensus regarding a clinically admissible disparity is lacking, few researchers have proposed that a 50-120 µm gap is clinically acceptable, whereas others have recommended gaps of less than 100 µm [5]. Therefore, it is important to minimize marginal gaps to decrease the incidence of associated complications.

This systematic review aimed to evaluate and compare the marginal adaptation of veneers using different fabrication methods, namely, conventional feldspathic porcelain laminate veneers (PLVs), computer-aided design-computer-aided machining (CAD-CAM) veneers, and pressed veneers.

## Review

Methodology

**Figure 1:**
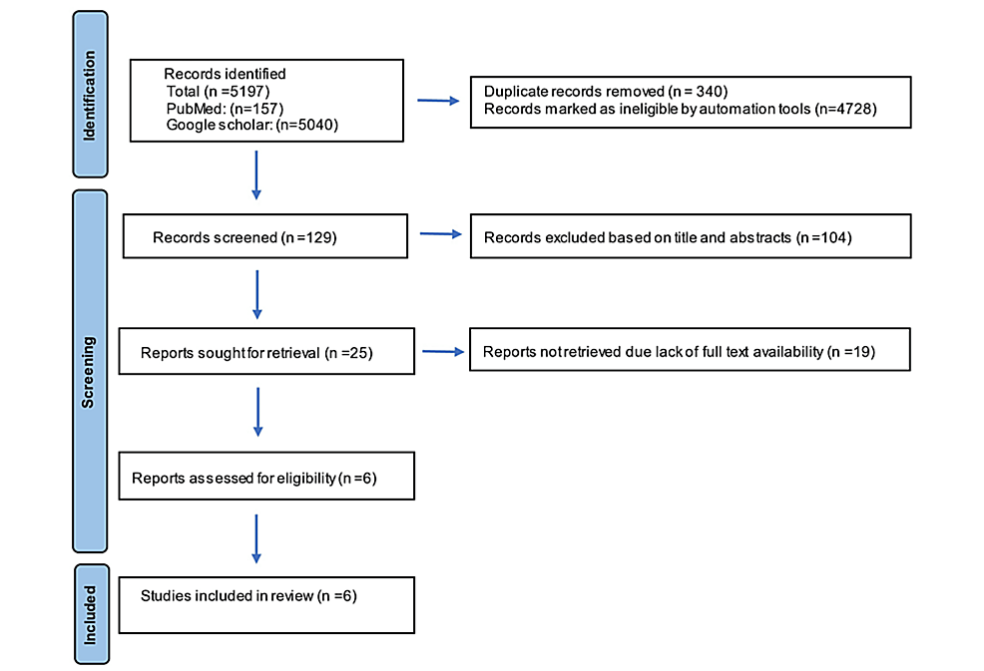
Flow chart of the search strategy used in this systematic review.

This systematic review followed the Preferred Reporting Items for Systematic Review and Meta-Analyses (PRISMA) guidelines and Population, Intervention, Comparison, Outcome (PICO) criteria. The PICO referred to veneers (P) fabricated with the CAD-CAM system and pressed ceramics (I) compared to the conventional feldspathic method present better marginal adaptation (O). PubMed and Google Scholar were explored for studies published between 1994 and 2020. The search strategy was a combination of medical subject heading terms “dental marginal adaptation.” “dental veneers,” “computer-aided design,” and “CAD-CAM” with the following text words “fit,” “gap,” “marginal,” “adaptation,” “accuracy,” “discrepancy,” “CAD,” “computer-aided,” “lithium silicate,” “feldspathic,” “leucite,” “milled,” and “composite.” All records identified were redeemed and imported into bibliographic software (Rayyan). Duplicates were removed. The entire search process is depicted in Figure 1.

Inclusion Criteria

In vitro and clinical prospective studies which investigated the marginal adaptation of CAD-CAM or heat-pressed veneers or conventional veneers were included in this review.

Exclusion Criteria

Case reports, case series, technique articles, abstracts, retrospective studies, review articles, studies only on composite veneering, and studies not published in the English language were excluded.

Data Extraction and Analysis

Two reviewers independently assessed all titles and abstracts according to the inclusion and exclusion criteria. The evaluation was performed jointly by both reviewers for the first 15 articles. Subsequently, the two reviewers continued with the evaluation independently. To avoid bias any studies identified by either reviewer during the initial screening were included. The full text of these selected studies was obtained for second-stage screening and submitted for data extraction. Information collected included journal name, year of publication, author names, type of preparation, type of porcelain, cementation, adaptation device, fabrication technique, follow-up period, type of study, and results (Table 1).

**Table 1:** Data extraction and analysis of the studies included in this systematic review. CAD-CAM: computer-aided design-computer-aided machining; PLV: porcelain laminate veneer; SEM: scanning electron microscopy

Journal, year	Author	Type of preparation	Type of porcelain	Cementation	Adaptation device	Fabrication technique	Follow up	Type of the study	Result
Journal of Dentistry, 2012	Lin et al. [6]	Full veneer and traditional veneer	Leucite-reinforced ceramic veneer and conventional sintered feldspathic porcelain veneer	Clear self-cured acrylic resin	Keyence digital microscope at 200×	CAD-CAM and conventional feldspathic	-	In vitro	Traditional veneers designed with ProCAD porcelain demonstrated lesser horizontal gaps
Journal of Dentistry, 2012	Aboushelib et al. [7]	Incisal lap preparation	Ceramic laminate veneers	Resin cement	SEM and stereomicroscope	CAD-CAM and pressed PLVs	60 days	In vitro	Pressable ceramic laminate veneers exhibited superior marginal fidelity, uniform and thinner cement film thickness, and lesser microleakage in contrast to machinable ceramic veneers
Journal of Prosthodontics, 2013	Jha et al. [8]	Window preparation	Refractory die technique, low-fusing feldspathic porcelain (IPS e. max Ceram), and lithium disilicate-reinforced glass ceramic (IPS e. max Press)	Dual-cure composite resin	SEM at 200× magnification	Conventional feldspathic and pressing technique	Seven days and three months	In vivo	Veneers of both groups showed a comparable marginal fidelity at the microscopic level at seven days and three months after cementation
Dental Research Journal, 2016	Ghaffari et al. [9]	Incisal overlap preparation	Feldspathic laminate system (Du Ceram LFC) and in Ceram laminate veneer	Hueless glue	Stereomicroscope at 46× magnification	Refractory die technique and slip cast technique	-	In vitro	PLVs fabricated with Inceram had a marginal gap within an acceptable range
Journal of Prosthetic Dentistry, 2018	Al-Dwairi et al. [10]	Full veneer preparation	Feldspathic glass ceramic, fine structure feldspar ceramic	Composite resin cement and variolink – N	SEM at 200× magnification	Pressed PLVs and CAD-CAM milling	-	In vitro	No statistically significant difference was found in gap measurements
Journal of Prosthodontics, 2017	Yuce et al. [11]	Incisal overlap preparation	Nano-fluor apatite glass ceramic	Adhesive luting cement	Light optical microscopy at 40× magnification	CAD-CAM (cerec) and heat-pressed (E. Max press)	6, 12, 18, and 24 months	In vivo	Marginal and internal adaptation of veneers were similar and within clinically acceptable ranges

Results

The electronic search identified 5,197 articles that were transferred to the Rayyan software, and 4,728 articles were marked as ineligible. After removing duplicate articles, 129 articles were included. Of these, 104 articles were excluded based on title and abstracts. As the full text was not available 19 articles were excluded as they were not available to download, and, finally, six articles were included in this review. Details of the search strategy are presented in the PRISMA flow chart. Out of the six studies included, four were in vitro and two were in vivo. All six articles evaluated CAD-CAM veneers, three evaluated pressed veneers, and three evaluated conventional feldspathic veneers. Marginal adaptation was evaluated by scanning electron microscopy in three studies and stereomicroscope in another three studies.

Discussion

Anterior PLVs offer a viable treatment option for the management of esthetic conditions with minimal tooth preparation. The physical and mechanical properties of the veneer material, its adhesion to the tooth structure, and marginal integrity play a vital role in the clinical success of PLVs. Ample marginal adaptation is key to avoiding excessive gaps, which, in turn, can lead to leakage, dissolution of the luting agent, secondary caries, and failure of the restoration. According to this review, the marginal adaptation of pressed and milled PLVs was similar.

PLVs are traditionally fabricated using a layering technique that uses refractory dies to support the condensed layers of the ceramic slurry. This maneuver gives the ceramist complete command over the layers incorporated, resulting in a natural-appearing restoration. Conversely, it needs time and labor to produce precisely fitting restorations. The entire fabrication procedure is highly technique sensitive [12]. A new generation of ceramic materials was introduced to dentistry using different techniques such as CAD-CAM and pressing technology. Pressable ceramics are fabricated by burning out wax patterns using the conventional lost wax technique and melting and pressing ceramic ingots under controlled pressure, temperature, and vacuum using computer-programmed press ovens. These ovens are equipped with a pneumatic press that activates an alumina plunger used to compress molten ceramic ingots. Press-on ceramics allow accurate reproduction of the anatomical features carved in the wax pattern and controlled processing of the ceramic material resulting in an accurate restoration with minimal internal structural defects. Nowadays, CAD-CAM requires nothing more than a few keyboard clicks to design and fabricate accurate restorations. Nevertheless, the shade and color of machinable ceramic-produced ceramic veneers are limited by the color of the selected block used to mill these restorations.

External marginal adaptation of ceramic veneers, which is defined as the vertical distance between the finish line of the prepared tooth and the margins of the fabricated veneers, plays an important role in their success [13]. The differences between the mean marginal gap values of PLVs could be because of variations in the preparation design, fabrication technique, restoration thickness, design complexity, geometry, many PLV designs (window preparation or butt joint preparation or minimal to no preparation designs), adhesive luting agent, and marginal fit measurement method [14].

In this review, six articles were included, of which three used scanning electron microscopy for fit evaluation and two used stereomicroscopes. Microscopy permitted the two-dimensional evaluation of marginal gaps at tooth and veneer junctions.

Of the six articles, three compared the marginal adaptation of CAD-CAM veneers with conventional feldspathic veneers and reported different conclusions. Lin et al. found the conventional feldspathic method to have better results [6]. Jha et al. concluded that both techniques showed similar results [8]. Ghaffari et al. showed that CAD-CAM produced better results when compared to the conventional method [9]. In CAD-CAM, these marginal gaps may be from overgrinding and chipping thin porcelain margins due to the fragile nature of the material and the vibrations caused by milling.

Studies conducted by Aboushelib et al. and Al-Dwairi et al showed that pressed ceramics exhibited better marginal adaptation than CAD-CAM veneers [7,10]. However, in the in vivo study reported by Yuce et al., the fabrication method, whether CAD-CAM or heat-pressed, had no effect on the marginal and internal adaptation of PLVs; however the values were higher compared to the above in vitro studies [11].

It has been proven that preparation with butt joint design produces better marginal adaptation than the palatal chamfer with milled PLVs. The variability could be due to the complex geometry, higher curvature, and thinner incisal edge found with the palatal chamfer than with the butt joint design, negatively influencing the scanning and milling procedure and leading to larger marginal and internal gap discrepancies.

According to previous studies, marginal adaptation values of restorations should be between 100 and 120 µm to avoid cement wear. Other studies reported that an acceptable marginal adaptation value varied in clinical conditions, and up to 300 µm was accepted for ceramic restorations [11]. All studies included in this review, irrespective of the type of veneer, had marginal gaps within the acceptable range, though no further conclusions could be drawn.

## Conclusions

The marginal fidelity of ceramic veneers issuing from the various fabrication techniques in this review, namely, conventional feldspathic veneers, CAD-CAM, and heat-pressed veneers, was found to be clinically acceptable. Feldspathic veneers exhibited better marginal adaptation compared to CAD-CAM veneers. However, between CAD-CAM and pressed veneers, varying results were observed. Because of limited literature, it was not feasible to establish a ranking of the different systems or conduct a proper comparison.
